# Reproducibility of isokinetic measures of the knee and ankle muscle strength in community-dwelling older adults without and with Alzheimer’s disease

**DOI:** 10.1186/s12877-022-03648-6

**Published:** 2022-12-07

**Authors:** Marcos Paulo Braz de Oliveira, Letícia Bojikian Calixtre, Paula Regina Mendes da Silva Serrão, Tatiana de Oliveira Sato, Anielle Cristhine de Medeiros Takahashi, Larissa Pires de Andrade

**Affiliations:** 1 Healthy Aging Research Laboratory, Physical Therapy Department, Federal University of São Carlos, Washington Luis Highway, Km 235, São Paulo São Carlos, Brazil; 2 Clinical and Occupational Kinesiology Laboratory, Physical Therapy Department, Federal University of São Carlos, São Carlos, Brazil; 3 Rheumatology and Hand Rehabilitation Research Laboratory, Physical Therapy Department, Federal University of São Carlos, São Carlos, Brazil; 4 Preventive Physical Therapy and Ergonomics Laboratory, Physical Therapy Department, Federal University of São Carlos, São Carlos, Brazil

**Keywords:** Alzheimer disease, Dementia, Muscle strength, Aging, Reproducibility of results

## Abstract

**Background:**

To interpret changes of muscle strength in older adults with Alzheimer’s disease (AD), determining the reliability of outcome measures is necessary. Therefore, the purpose of the present study was to investigate the relative and absolute intra-rater reliability of concentric isokinetic measures of the knee and ankle muscle strength in community-dwelling older adults without and with AD in the mild and moderate stages.

**Methods:**

A methodological study was conducted. The participants were submitted to two isokinetic evaluations with an interval of three to seven days. The evaluations consisted of knee extension and flexion at 60°/s (five repetitions) and 180°/s (15 repetitions) and plantar flexion and dorsiflexion of the ankle at 30°/s (five repetitions). The measures of interest were peak torque, average peak torque and total work. The intraclass correlation coefficient two-way mixed model of a single-measure (ICC_3,1_), standard error of measurement (SEM) and minimal detectable change at the 95% confidence interval (MDC_95_) were calculated. The ICC_3,1_ was interpreted based on Munro’s classification. Standard error of measurement and MDC_95_ were analyzed in absolute and relative values (percentage of error [SEM%] and change [MDC_95_%]).

**Results:**

A total of 62 older adults were included and allocated to the three groups: mild-AD (n = 22, 79.9 years, 15 female and seven male), moderate-AD (*n* = 20, 81.6 years, 15 female and five male) and without-AD (*n* = 20, 74.3 years, 10 female and seven male). The ICCs_3,1_ of the measures of knee were high/very high in the three groups (0.71–0.98). The ICCs_3,1_ of the measures of ankle were high/very high in the mild-AD group (0.78–0.92), moderate/high/very high in the moderate-AD group (0.63–0.93) and high/very high in the group without-AD (0.84–0.97). The measurements of knee extensors at 60°/s, knee extensors (peak torque and total work), with the exception of peak torque in the mild-AD group, and flexors (average peak torque) at 180°/s, and ankle dorsiflexors at 30°/s had the lowest of SEM% and MDC95% in the three groups.

**Conclusion:**

Concentric isokinetic measures are reliable for the assessment of knee and ankle muscle strength in community-dwelling older adults without and with AD in the mild and moderate stages.

## Introduction

Alzheimer’s disease (AD) is the most common cause of dementia, accounting for 60 to 80% of cases, and the incidence and prevalence of the disease increases with age [1]. Alzheimer’s disease is characterized by the loss of recent memory, cognitive changes and dependence regarding the performance of activities of daily living [1]. Older adults affected with AD also have functional limitations, such as deficits in postural balance [2], a reduced gait speed [3], and diminished muscle strength [4].

Clinical and laboratory-based tests can be used for the assessment of lower limb muscle strength as well as the identification of improvement, maintenance or decline. Clinical tests have proven reliable for older adults with AD, such as the Five-times and 30-second Sit to Stand test [5]. However, laboratory-based tests for this population are scarce. To date, only the hand-held dynamometer has proven reliability for the assessment of knee muscle strength in older adults with AD [6], and knee and ankle muscle strength in older adults with dementia [7].

An isokinetic dynamometer is an instrument used to evaluate neuromuscular function through isokinetic measures, such as maximum muscle strength (peak torque), the average of maximum muscle strength (average peak torque) and the capacity of the muscle group to maintain maximum muscle strength (total work) [8–10]. Some isokinetic measures are related to functional performance in healthy older adults. For instance, lower peak knee extensor torque is associated with deficits in dynamic balance as well as reductions in mobility and lower limb muscle strength [11]. Thus, torque is a representative measure for the older population.

Previous studies have determined the reliability of isokinetic dynamometry in healthy older adults [12–15]. However, the reliability of the isokinetic measures has not been investigated in older adults with AD. Although isokinetic dynamometry is considered the gold standard for the assessment of muscle strength [8–10], the hand-held dynamometer has been used more due to its lower complexity and cost in comparison to the isokinetic dynamometer. The adaptation and standardization of the isokinetic assessment in this study will enable the use of a isokinetic dynamometer in future studies with this population.

Moreover, identifying reliable methods for the assessment of knee and ankle muscles is important. Knee extensors and flexors are related to the tasks of sitting and standing [16, 17], and older adults with AD have greater difficulty performing these tasks in comparison to healthy older adults [18]. Plantar flexors and dorsiflexors are related to the occurrence of falls [19], and older adults with AD have a greater incidence of falls compared to healthy older adults [20].

Although the isokinetic dynamometer has been used of the evaluation of knee extensors and flexors in a physical intervention study involving older adults with AD [21], no previous reliability studies have been conducted involving the isokinetic dynamometer for this population. Moreover, older adults with AD may have difficulties understanding and following verbal commands [22], which could hinder the performance of the test and lower the reliability of the measure. It is therefore necessary to investigate whether isokinetic measures are reliable for the assessment of lower limb muscle strength in older adults with AD. The purpose of the present study was to investigate relative and absolute intra-rater reliability of concentric isokinetic measures (peak torque, average peak torque and total work) of the knee and ankle muscle strength in community-dwelling older adults without and with AD in the mild and moderate stages.

## Methods

### Study design and ethics

A methodological study was conducted at the Healthy Aging Research Laboratory and Isokinetic Dynamometry Laboratory of the Physical Therapy Department of Federal University of São Carlos (UFSCar), São Paulo, Brazil. This study was approved by Research Ethics Committee for Human Beings of the UFSCar (certificate number: 88921118.4.0000.5504). The study was conducted according to the guidelines of the Declaration of Helsinki. Verbal and written informed consent was obtained from all participants. In the groups of participants with Alzheimer’s disease, the caregivers or legally authorized representatives gave informed consent in name of their care recipients and verbal informed consent was obtained from the participants on the day of the evaluations. Moreover, the caregiver or legally authorized representatives of illiterate participants provided informed consent for the study. The data were collected between February and December 2019. The checklist of the Guidelines for Reporting Reliability and Agreement Studies (GRRAS) were followed [23, 24].

### Participants

Eligible participants were community-dwelling older adults without and with AD, ≥ 65 years of age, both sexes and with no musculoskeletal disorders of the knee or ankle, such as fractures, pain, osteoporosis or previous surgeries. The participants were recruited from the Health School Unit of UFSCar, Open University for Older Adults and Family Health Programs in the city of São Carlos.

The inclusion criteria for the older adults with AD were (1) medical diagnosis of AD based on the Diagnostic and Statistical Manual of Mental Disorders (DSM-V) [25], (2) being classified in the mild or moderate stages of AD based on the Clinical Dementia Rating (CDR) scale [26, 27], and (3) score below the cutoff point for the detection of dementia on the Mini Mental State Examination (MMSE) adjusted for years of education: 20 (illiterate), 25 (1 to 4 years of education), 26.5 (5 to 8 years of education), 28 (9 to 11 years of education) and 29 (≥ 12 years of education) [28, 29], and, (4) being clinically stable. The exclusion criteria for the older adults with AD were (1) comprehension difficulties (e.g., inability to state one’s own name, hand over or receive an object when requested to do so, etc.), (2) dementia of other etiologies (e.g., Lewy body, vascular, frontotemporal, etc.), (3) other neurodegenerative diseases besides AD (e.g., Parkinson’s disease) or non-neurodegenerative diseases (e.g., stroke), and (4) diagnosis of depression.

The medical diagnosis of AD was performed by geriatricians or neurologists based on the DSM-V [25]. The classification of AD stage was performed by the researchers based on the CDR, who have amply experience proved by previous studies [21, 30, 31]. This scale quantifies the severity of dementia and is composed of six domains: memory, orientation, judgment and problem solving, community affairs, home and hobbies and personal care. The final score is used to classify very mild (CDR = 0.5), mild (CDR = 1), moderate (CDR = 2) and severe (CDR = 3) dementia [26, 27].

The inclusion criteria for the older adults without-AD were (1) preserved cognition, with score above the cutoff point on the MMSE adjusted for years of education [28, 29], (2) not meeting the criteria for Mild Cognitive Impairment or dementia (e.g., AD) [25, 32], and (3) being clinically stable. The exclusion criteria were (1) neurodegenerative and non-neurodegenerative diseases, and (2) diagnosis of depression.

### Clinical and demographic measures

Global cognitive function was assessed using the MMSE, which addresses orientation, memory, language and visuospatial skills. The maximum score is 30 points, with higher scores indicating a better performance [28, 29]. Grip strength was measured using the JAMAR® Hydraulic Hand Dynamometer (Model PC-5030J1, Fred Sammons, Inc., Burr Ridge, IL, USA). The participants were instructed to use the greatest possible strength and maintain the contraction for six seconds. Three readings were taken on the dominant hand and the average was calculated, with higher scores indicating a better performance [33]. Depressive symptoms were assessed using the Geriatric Depression Scale, which is composed of 15 affirmative or negative questions (yes = presence of symptom; no = absence of symptom), with a score > 5 indicating the presence of depressive symptoms [34, 35]. Physical activity level was assessed using the Modified Baecke Questionnaire (MBQ), which is composed of 10 items related to activities of daily living as well as the investigation of free time and physical activity, with lower scores indicating a lower physical activity level [36, 37]. For those with AD, the MBQ was administered to the caregiver to obtain information on the participant. A caregiver was considered a family member or guardian who spent at least half of the day with the older person four times a week. The procedures for the application of the tests of the clinical measures followed the orientations recommended in the original version.

### Isokinetic evaluations

The isokinetic evaluations were performed using the Biodex System isokinetic dynamometer (Biodex Multi Joint System PRO, Shirley, New York, USA) with a sampling frequency of 100 Hz.

### Description of isokinetic evaluation of knee and ankle muscle strength

The isokinetic evaluations of knee extension and flexion were performed with angular velocities of 60º/s (five maximum voluntary repetitions) and 180°/s (15 maximum voluntary repetitions) in a total range of 70º starting from 90° knee flexion (0° = complete extension). The isokinetic evaluations of ankle plantar flexion and dorsiflexion were performed with an angular velocity of 30º/s (five maximum voluntary repetitions) in a total range of 45º starting from 35° of plantar flexion to 10º of dorsiflexion (0° = neutral position).

As an unprecedented study involving older adults with AD, the parameters of the isokinetic evaluations were based on previous reliability studies conducted with healthy older adults [13, 14]. The capacity of the knee extensors and flexors to generate maximum force seems to occur at low and high velocities (60°/s and 180°/s) in healthy older adults. The production of maximum plantar flexor and dorsiflexor strength in healthy older adults seems to occur at low velocities (30°/s), which justifies the choice of the angular velocities used in the present study [38, 39].

The participants underwent evaluations in the sitting position on the isokinetic dynamometer. The positioning and procedures for the collection of data were performed following the specifications of the manufacturer [40]. The isokinetic evaluations of the knee and ankle muscle strength were performed with the dominant lower limb. To determine dominance, the participants were asked to kick a ball with a much strength as possible. The isokinetic measures used for the analyses were peak torque, average peak torque and total work; higher values of these measures indicate a better performance. A comprehensive evaluation of maximum muscle strength should include these three measures. Peak torque and average peak torque were normalized by individual body mass (isokinetic measure / body mass (kg) × 100).

### Adaptation and standardization of isokinetic evaluations

The adaptation and standardization of the isokinetic evaluations were ensured with communication strategies adopted for the participants with AD, such as maintaining eye contact when speaking, speaking slowly and clearly, explaining the actions prior to their execution as well as repeating the explanation of the correct execution and demonstrating the tests. Prior to the isokinetic evaluations on D1 and D2, an explanation was given and the tests were demonstrated on the non-dominant lower limb, followed by familiarization on the dominant lower limb for each test and angular velocity with three submaximal repetitions and one maximum repetition [9–11]. After familiarization, the main examiner asked the participants if they understood the execution of the tests. The evaluations were initiated after verbal confirmation from the participants that they understood the instructions of the tests. After three minutes of rest, the isokinetic evaluation was performed. During the evaluations, standardized, vigorous verbal commands were given: Ready, set, go! (the word “go” was repeated throughout the time of the contractions). During the three five-minute rest intervals, the explanation of the correct execution of the tests was repeated and the participants verbally confirmed that they had understood the explanation. These procedures were adopted due to the limitations of older adults with AD (e.g., difficulties in communication and the comprehension of verbal commands). During the isokinetic evaluations, heart rate, blood oxygen saturation and blood pressure were monitored to ensure the safety of the older adults. For the purposes of comparison, the isokinetic evaluations were the same for all three groups (mild-AD, moderate-AD and without-AD).

### Procedures

The evaluations were conducted on two days (D1 = test; D2 = retest) with a minimum interval of three days for muscle recovery and a maximum of seven days [41, 42]. On D1, clinical and demographic data were collected, followed by the isokinetic evaluation of the knee and ankle muscle strength in random order. On D2, the isokinetic evaluation was performed again in the same order as that used on D1. The isokinetic evaluations were performed by a single examiner. The examiner on D2 was blinded to the results of the tests on D1, as the data were not exported from the isokinetic dynamometer. Every effort was made to maintain the factors related to the evaluation sessions consistent (same time of the day [morning or afternoon] and the same members of the team assisting in the evaluations).

### Statistical analysis

The sample size was determined *a priori* using the method proposed by Walter, Eliasziw and Donner (1998) [43]. Based on this method, a sample of 22 participants per group was needed to achieve an 80% test power, considering a 5% significance level, acceptable intraclass correlation coefficient (ICC) of 0.3 and ICC of 0.70 for the two measures (test and retest).

The normality and homoscedasticity of the data were analyzed using the Shapiro-Wilk and Levene tests, respectively. The clinical and demographic characteristics of the participants allocated to the mild-AD, moderate-AD and without-AD groups were compared using one-way ANOVA and Tukey’ post hoc test (parametric variables), considering *p* < 0.05; Kruskal-Wallis and Mann-Whitney test with the Bonferroni correction (nonparametric variables), considering *p* < 0.016; and chi-square test (categorical variables), considering *p* < 0.016.

Relative reliability was determined using the ICC two-way mixed model of a single-measure (ICC_3,1_) at the 95% confidence interval. The ICC_3,1_ was interpreted based on the Munro’s classification (very low: 0.00-0.25, low: 0.26–0.49, moderate: 0.50–0.69, high: 0.70–0.89 and very high: 0.90-1.00) [44, 45]. Absolute reliability was determined by the standard error of measurement (SEM) and minimal detectable change at the 95% confidence interval (MDC_95_). Standard error of measurement and MDC_95_ values were interpreted based on absolute and relative values. Absolute values were calculated using the following equations: SEM = SD√1-ICC and MDC_95_ = SEM×√2 × 1.64 [46]. Relative values were reported in percentage of error (SEM%) and change (MDC_95_%). A SEM% value ≤ 10% and MDC% value ≤ 30% were considered acceptable [47]. Relative values were calculated using the following formulas: SEM% = SEM / mean of measures x 100% and MDC_95_% = MDC_95_ / mean of measures x 100% [46]. The mild-AD, moderate-AD and without-AD groups were considered separately in the reliability analyses. The data were managed and analyzed using SPSS 23 (IBM; Chicago, IL, USA) and Excel 2010 (Microsoft Corporation, Redmond, Washington, USA).

## Results

**Fig. 1:**
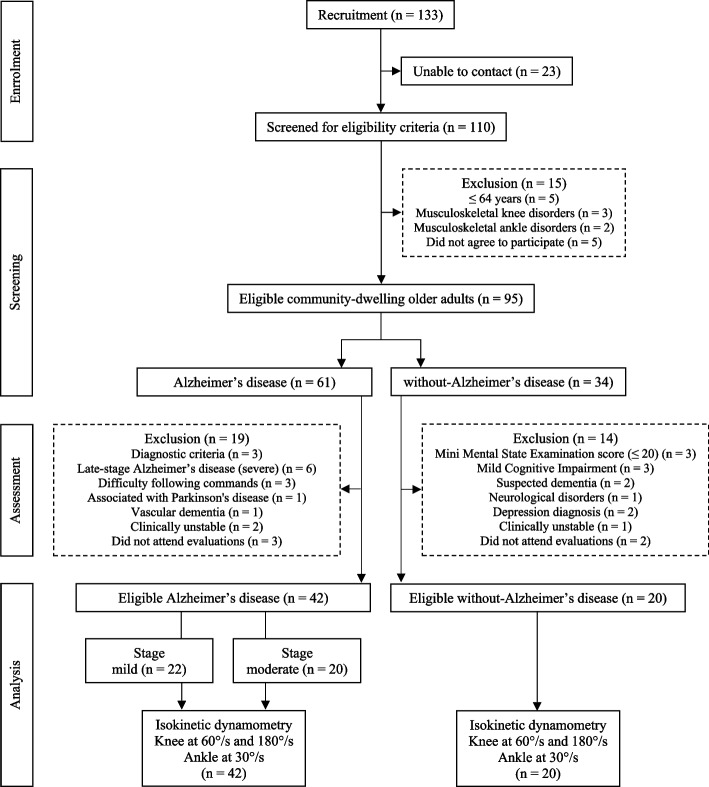
Flow diagram of participants

A total of 62 older adults were included and allocated to the three groups: mild-AD (*n* = 22), moderate-AD (*n* = 20) and without-AD (*n* = 20). All older adults performed the proposed evaluations. No harmful or undesirable effects occurred during the isokinetic evaluations (D1 and D2). On D2, the participants reported no symptoms of fatigue or muscle pain related to D1. The participants were contacted by telephone 24 to 72 h after D2 and no discomfort was reported. All participants followed the instructions and executed the isokinetic tests correctly with the adequate range of motion. Therefore, one may infer that the participants understood the adapted, standardized instructions given by the examiner. The isokinetic evaluations were performed by a single examiner (physiotherapist with three years of experience in evaluations involving an isokinetic dynamometer and six years of experience with physical and cognitive evaluations and treatment with physical exercise for older adults with AD). Figure 1 displays the flowchart of the participants in the study.

The clinical and demographic characteristics of the three groups are displayed in Table 1. Similarities were found among the groups in terms of sex, weight, height, body mass index, lower limb dominance and depressive symptoms. As expected, significant differences were found regarding some characteristics. The moderate-AD group had an older mean age compared to the group without-AD (*p* = 0.001). Cognitive function differed significantly among the three groups, with the moderate-AD group achieving the worst performance on the MMSE (*p* = 0.001). The mild-AD and moderate-AD groups had a lower physical activity level (*p* = 0.012; *p* = 0.007) and grip strength (*p* = 0.002;

*p* = 0.001) compared to the group without-AD.

**Table 1:** Clinical and demographic characteristics of participants

Variables	All participants (*n* = 62)	mild-AD (CDR = 1) (*n* = 22)	moderate-AD (CDR = 2) (*n* = 20)	without-AD (*n* = 20)	Difference between three groups *(p-value)*	**Post-hoc analysis *(p-value)*		
						mild-AD *versus* without-AD	moderate-AD *versus* without-AD	mild-AD *versus* moderate-AD
Age, years,$\stackrel{-}{\text{X}}$(SD) (range)	77.9 (6.1) (66–91)	77.9 (5.3) (66–86)	81.6 (6.2) (67–91)	74.3 (4.5) (66–84)	0.001*	0.093	0.001*	0.074
Sex, n (%)	40 F / 22 M	15 F / 7 M	15 F / 5 M	10 F / 10 M	0.231	-	-	-
Weight, kg,$\stackrel{-}{\text{X}}$(SD)	66.3 (11.6)	65.1 (12.3)	63.7 (12.0)	70.1 (9.8)	0.185	-	-	-
Height, m,$\stackrel{-}{\text{X}}$(SD)	1.6 (0.1)	1.6 (0.1)	1.5 (0.1)	1.6 (0.1)	0.139	-	-	-
BMI, kg/m^2^,$\stackrel{-}{\text{X}}$(SD)	26.3 (3.5)	25.8 (3.8)	26.2 (3.3)	26.8 (3.4)	0.649	-	-	-
Lower limb dominance, n, right/left	57 R / 5 L	20 R / 2 L	18 R / 2 L	19 R / 1 L	0.825	-	-	-
MMSE (0–30),$\stackrel{-}{\text{X}}$(SD) (range)	21.8 (5.3) (9–30)	21.7 (2.5) (17–25)	16.0 (4.0) (9–25)	27.7 (1.5) (25–30)	0.001*	0.001*	0.001*	0.001*
Grip strength, kgf,$\stackrel{-}{\text{X}}$(SD)	23.0 (8.9)	21.9 (7.5)	18.2 (8.0)	29.1 (8.2)	0.001*	0.002*	0.001*	0.151
GDS (0–15),$\stackrel{-}{\text{X}}$(SD)	2.8 (2.4)	3.8 (2.7)	2.5 (1.9)	2.0 (2.1)	0.047*	0.019	0.252	0.133
MBQ,$\stackrel{-}{\text{X}}$(SD)	5.7 (4.6)	4.5 (3.2)	4.2 (3.3)	8.5 (5.7)	0.010*	0.012*	0.007*	0.678

*Abbreviations:AD* Alzheimer’s disease, *CDR* Clinical Dementia Rating, *MMSE* Mini Mental State Examination, *MBQ* Modified Baecke Questionnaire, *GDS* Geriatric Depression Scale, *BMI* Body Mass Index, *n* Number of participants, $\stackrel{-}{\text{X}}$ Mean, *SD* Standard deviation, *F* Female, *M* Male, *R* Right, *L* Left, *kg* Kilogram, *m* Meter, *kg/m2* Kilogram per meter square, *kgf* Kilogram-force

*Statistically significant diferences (*p* < 0.05); **Post hoc analyses were performed for clinical and demographic variables that presented statistically significant differences (*p* < 0.05)

### Isokinetic measures of knee muscle strength

The reliability values of the isokinetic measures of the knee muscle strength at 60°/s are displayed in Table 2. For relative reliability, ICCs_3,1_ were high or very high (0.71–0.92) for all measures in the three groups. For absolute reliability, SEM values ranged from 7.5 to 26.7 in the mild-AD group, 5.1 to 35.4 in the moderate-AD group and 5.9 to 33.8 in the group without-AD. The MDC_95_ values ranged from 17.3 to 61.9 in the mild-AD group, 11.9 to 82.0 in the moderate-AD group and 13.7 to 78.3 in the group without-AD. All knee extensor measures had lower SEM% and MDC_95_% in comparison to the flexors in all three groups.

**Table 2:** Intra-rater relative and absolute reliability of concentric isokinetic measures of knee extensor and flexor muscle strength at angular velocity of 60°/s

Participants	Measures	Knee at 60°/s					
		Peak torque (Nm/Kg)	Peak torque (Nm/Kg)	Average peak torque (Nm/Kg)	Average peak torque (Nm/Kg)	Total work (J)	Total work (J)
		Extension	Flexion	Extension	Flexion	Extension	Flexion
mild-AD (CDR = 1) (*n* = 22)	Session 1,$\stackrel{-}{\text{X}}$(SD)	83.2 (22.6)	34.5 (16.8)	75.2 (21.4)	29.2 (15.7)	211.8 (68.5)	78.4 (55.4)
	Session 2,$\stackrel{-}{\text{X}}$(SD)	88.7 (29.1)	42.5 (18.0)	79.9 (27.0)	36.7 (17.9)	225.2 (86.0)	100.3 (62.4)
	ICC_3,1_ (CI_95_)	0.71 (0.43–0.87)	0.71 (0.30–0.88)	0.86 (0.67–0.94)	0.81 (0.47–0.93)	0.88 (0.72–0.95)	0.84 (0.58–0.94)
	SEM	13.8	9.5	9.1	7.5	26.7	23.4
	SEM%	15.6%	22.3%	11.4%	20.4%	11.8%	23.3%
	MDC_95_	32.1	21.9	21.0	17.3	61.9	54.4
	MDC_95_%	36.2%	51.5%	26.3%	47.1%	27.5%	54.2%
moderate-AD (CDR = 2) (*n* = 20)	Session 1,$\stackrel{-}{\text{X}}$(SD)	73.1 (33.0)	32.1 (16.4)	64.2 (30.0)	26.7 (14.2)	172.7 (101.3)	71.4 (50.6)
	Session 2,$\stackrel{-}{\text{X}}$(SD)	66.1 (30.3)	30.8 (20.4)	58.2 (26.6)	25.8 (18.2)	162.5 (83.8)	69.6 (63.7)
	ICC_3,1_ (CI_95_)	0.81 (0.58–0.92)	0.80 (0.56–0.91)	0.87 (0.68–0.95)	0.90 (0.74–0.96)	0.85 (0.63–0.94)	0.88 (0.70–0.95)
	SEM	13.8	8.2	10.1	5.1	35.4	19.5
	SEM%	18.9%	25.5%	15.7%	19.1%	20.5%	27.3%
	MDC_95_	31.9	19.1	23.5	11.9	82.0	45.2
	MDC_95_%	43.6%	59.5%	36.6%	44.6%	47.5%	63.3%
without-AD (*n* = 20)	Session 1,$\stackrel{-}{\text{X}}$(SD)	115.8 (23.4)	54.6 (24.6)	104.0 (23.8)	49.7 (24.0)	321.4 (72.8)	150.2 (82.7)
	Session 2,$\stackrel{-}{\text{X}}$(SD)	120.7 (27.2)	60.6 (20.3)	110.8 (27.4)	53.9 (18.4)	336.2 (86.3)	171.2 (74.5)
	ICC_3,1_ (CI_95_)	0.73 (0.44–0.82)	0.87 (0.64–0.95)	0.79 (0.49–0.91)	0.92 (0.80–0.97)	0.82 (0.55–0.93)	0.92 (0.76–0.97)
	SEM	13.1	8.1	11.6	5.9	33.8	22.4
	SEM%	10.8%	13.4%	10.5%	10.9%	10.0%	13.1%
	MDC_95_	30.4	19.9	27.0	13.7	78.3	52.0
	MDC_95_%	25.2%	32.8%	24.4%	25.4%	23.3%	30.4%

The ICC_3,1_ was interpreted based on the Munro’s classification (very low: 0.00-0.25, low: 0.26–0.49, moderate: 0.50–0.69, high: 0.70–0.89 and very high: 0.90-1.00); A SEM% value ≤ 10% and MDC% value ≤ 30% were considered acceptable

Equations and formulas: SEM = SD√1-ICC (absolute values); SEM% = SEM / mean of measures x 100% (relative values); MDC_95_ = SEM×√2 × 1.64 (absolute values); MDC_95_% = MDC_95_ / mean of measures x 100% (relative values)

*Abbreviations: AD* Alzheimer’s disease, *CDR* Clinical Dementia Rating, *ICC*_3,1_ Intraclass correlation coeficiente two-way mixed model of a single-measure, *CI*_95_ 95% confidence interval, *SEM* Standard error of measurement, *SEM%* Percentage of error, *MDC*_95_ Minimal detectable change at the 95% confidence interval, *MDC*_95_% Percentage of change, *n* Number of participants, $\stackrel{-}{\text{X}}$ Mean, *SD* Standard deviation, *Nm/Kg* Newton metre per kilogram, *J* Joules, *°/s* Degree per second

The reliability values of the isokinetic measures of the knee muscle strength at 180°/s are displayed in Table 3. For relative reliability, ICCs_3,1_ were high or very high (0.75–0.98) for all measures in the three groups. For absolute reliability, SEM values ranged from 2.4 to 45.5 in the mild-AD group, 3.0 to 41.4 in the moderate-AD group and 3.5 to 59.6 in the group without-AD. The MDC_95_ values ranged from 5.6 to 105.6 in the mild-AD group, 7.0 to 96.0 in the moderate-AD group and 8.1 to 138.2 in the group without-AD. The knee extensor measures (peak torque and total work) had lower SEM% and MDC_95_% in comparison to the knee flexors in the three groups, with the exception of peak torque in the mild-AD group. The knee flexor measures (average peak torque) had lower SEM% and MDC_95_% in comparison to the knee extensors in the three groups.

**Table 3:** Intra-rater relative and absolute reliability of concentric isokinetic measures of knee extensor and flexor muscle strength at angular velocity of 180°/s

Participants	Measures	Knee at 180°/s					
		Peak torque (Nm/Kg)	Peak torque (Nm/Kg)	Average peak torque (Nm/Kg)	Average peak torque (Nm/Kg)	Total work (J)	Total work (J)
		Extension	Flexion	Extension	Flexion	Extension	Flexion
mild-AD (CDR = 1) (*n* = 22)	Session 1, $\stackrel{-}{\text{X}}$(SD)	48.5 (18.8)	33.0 (16.1)	39.8 (16.5)	26.1 (15.2)	295.7 (135.3)	101.5 (121.7)
	Session 2, $\stackrel{-}{\text{X}}$(SD)	51.7 (18.6)	36.3 (18.2)	42.1 (16.3)	28.4 (16.1)	321.1 (145.1)	114.1 (139.4)
	ICC_3,1_ (CI_95_)	0.79 (0.56–0.90)	0.89 (0.75–0.96)	0.87 (0.68–0.95)	0.98 (0.93–0.99)	0.89 (0.75–0.95)	0.96 (0.91–0.98)
	SEM	8.5	5.5	5.9	2.4	45.5	25.2
	SEM%	16.4%	15.1%	14.0%	8.4%	14.2%	22.1%
	MDC_95_	19.8	12.9	13.7	5.6	105.6	58.5
	MDC_95_%	38.3%	35.5%	32.5%	19.7%	32.9%	51.3%
moderate-AD (CDR = 2) (*n* = 20)	Session 1, $\stackrel{-}{\text{X}}$(SD)	39.1 (13.4)	29.1 (11.5)	32.2 (12.9)	22.3 (11.5)	232.1 (114.9)	64.7 (63.1)
	Session 2, $\stackrel{-}{\text{X}}$(SD)	41.6 (12.9)	26.1 (10.3)	33.7 (9.9)	19.4 (9.1)	241.4 (109.7)	52.6 (75.9)
	ICC_3,1_ (CI_95_)	0.75 (0.48–0.89)	0.82 (0.58–0.93)	0.80 (0.49–0.92)	0.91 (0.75–0.97)	0.86 (0.65–0.94)	0.88 (0.70–0.95)
	SEM	6.5	4.6	5.1	3.0	41.4	24.0
	SEM%	15.6%	15.8%	15.1%	13.4%	17.1%	37.1%
	MDC_95_	15.1	10.7	11.9	7.0	96.0	55.6
	MDC_95_%	36.3%	36.8%	35.3%	31.4%	39.8%	85.9%
without-AD (*n* = 20)	Session 1, $\stackrel{-}{\text{X}}$(SD)	69.6 (16.6)	47.6 (20.9)	58.7 (13.8)	41.0 (19.7)	544.9 (153.4)	250.8 (204.9)
	Session 2, $\stackrel{-}{\text{X}}$(SD)	74.0 (20.8)	48.2 (17.3)	63.2 (18.2)	40.7 (16.2)	582.4 (193.7)	274.3 (211.7)
	ICC_3,1_ (CI_95_)	0.79 (0.54–0.91)	0.91 (0.78–0.96)	0.83 (0.58–0.93)	0.96 (0.90–0.98)	0.88 (0.70–0.95)	0.97 (0.92–0.99)
	SEM	8.6	5.7	6.6	3.5	59.6	36.8
	SEM%	11.6%	11.8%	10.4%	8.5%	10.2%	13.4%
	MDC_95_	20.0	13.3	15.3	8.1	138.2	85.5
	MDC_95_%	27.0%	27.6%	24.2%	19.7%	23.7%	31.2%

The ICC_3,1_ was interpreted based on the Munro’s classification (very low: 0.00-0.25, low: 0.26–0.49, moderate: 0.50–0.69, high: 0.70–0.89 and very high: 0.90-1.00); A SEM% value ≤ 10% and MDC% value ≤ 30% were considered acceptable

Equations and formulas: SEM = SD√1-ICC (absolute values); SEM% = SEM / mean of measures x 100% (relative values); MDC_95_ = SEM×√2 × 1.64 (absolute values); MDC_95_% = MDC_95_ / mean of measures x 100% (relative values)

*Abbreviations:* *AD* Alzheimer’s disease, *CDR *Clinical Dementia Rating, *ICC*_3,1_ Intraclass correlation coeficiente two-way mixed model of a single-measure, *CI*_95_ 95% confidence interval, *SEM *Standard error of measurement; *SEM%* Percentage of error, *MDC*_95_ Minimal detectable change at the 95% confidence interval, *MDC*_95_%Percentage of change, *n* Number of participants, $\stackrel{-}{\text{X}}$ Mean, *SD* Standard deviation, *Nm/Kg *Newton metre per kilogram, *J* Joules, *°/s* Degree per second

### Isokinetic measures of ankle muscle strength

The reliability values of the isokinetic measures of the ankle muscle strength at 30°/s are displayed in Table 4. For relative reliability, ICCs_3,1_ were high or very high (0.78–0.92) for all measures in the mild-AD group, moderate, high or very high (0.63–0.93) in the moderate-AD group and high or very high (0.84–0.97) in the group without-AD. For absolute reliability, SEM values ranged from 1.9 to 11.6 in the mild-AD group, 4.1 to 9.5 in the moderate-AD group and 6.6 to 29.2 in the group without-AD. The MDC_95_ values ranged from 4.5 to 26.9 in the mild-AD group, 9.5 to 22.1 in the moderate-AD group and 6.6 to 29.12 in the group without-AD. All ankle dorsiflexor measures had lower SEM% and MDC_95_% in comparison to the plantar flexors in all three groups.

**Table 4:** Intra-rater relative and absolute reliability of concentric isokinetic measures of plantar flexor and dorsiflexor muscle strength at angular velocity of 30°/s

Participants	Measures	Ankle at 30°/s					
		Peak torque (Nm/Kg)	Peak torque (Nm/Kg)	Average peak torque (Nm/Kg)	Average peak torque (Nm/Kg)	Total work (J)	Total work (J)
		Plantarflexion	Dorsiflexion	Plantarflexion	Dorsiflexion	Plantarflexion	Dorsiflexion
mild-AD (CDR = 1) (*n* = 22)	Session 1,$\stackrel{-}{\text{X}}$(SD)	43.9 (15.9)	31.7 (8.2)	35.4 (12.9)	29.4 (7.3)	41.8 (23.3)	45.1 (20.2)
	Session 2,$\stackrel{-}{\text{X}}$(SD)	48.4 (16.2)	31.5 (7.4)	38.5 (15.6)	29.0 (6.3)	45.6 (27.7)	44.2 (19.5)
	ICC_3,1_ (CI_95_)	0.78 (0.52–0.90)	0.78 (0.54–0.90)	0.83 (0.59–0.93)	0.92 (0.80–0.96)	0.79 (0.50–0.91)	0.91 (0.78–0.96)
	SEM	7.5	3.6	5.9	1.9	11.6	5.8
	SEM%	15.5%	11.3%	15.3%	6.5%	25.4%	12.9%
	MDC_95_	17.5	8.4	13.8	4.5	26.9	13.6
	MDC_95_%	36.1%	26.5%	35.8%	15.3%	59.0%	30.1%
moderate-AD (CDR = 2) (*n* = 20)	Session 1,$\stackrel{-}{\text{X}}$(SD)	37.2 (17.9)	31.7 (7.9)	30.2 (16.1)	27.8 (6.0)	35.2 (28.9)	39.6 (14.9)
	Session 2,$\stackrel{-}{\text{X}}$(SD)	38.4 (22.9)	30.9 (8.0)	31.4 (19.5)	28.6 (7.6)	34.6 (34.8)	40.3 (13.3)
	ICC_3,1_ (CI_95_)	0.78 (0.52–0.91)	0.66 (0.32–0.85)	0.93 (0.83–0.97)	0.63 (0.05–0.85)	0.93 (0.83–0.97)	0.63 (0.05–0.86)
	SEM	9.5	4.6	4.6	4.1	8.0	8.4
	SEM%	24.7%	14.5%	14.6%	14.3%	22.7%	20.8%
	MDC_95_	22.1	10.6	10.8	9.5	18.7	19.5
	MDC_95_%	57.5%	33.4%	34.4%	33.2%	53.1%	48.4%
without-AD (*n* = 20)	Session 1,$\stackrel{-}{\text{X}}$(SD)	70.6 (34.9)	41.9 (11.5)	62.6 (31.1)	37.4 (8.5)	108.7 (74.1)	70.4 (25.2)
	Session 2,$\stackrel{-}{\text{X}}$(SD)	76.7 (40.6)	44.0 (11.0)	67.8 (35.2)	39.9 (8.9)	117.3 (75.4)	75.9 (20.0)
	ICC_3,1_ (CI_95_)	0.92 (0.80–0.97)	0.84 (0.65–0.93)	0.96 (0.89–0.98)	0.89 (0.70–0.96)	0.97 (0.93–0.99)	0.87 (0.69–0.95)
	SEM	10.4	4.4	6.7	2.9	12.6	8.0
	SEM%	13.5%	10.0%	9.9%	7.3%	10.7%	10.5%
	MDC_95_	24.1	10.2	15.5	6.6	29.2	18.5
	MDC_95_%	31.4%	23.2%	22.9%	16.5%	24.9%	24.4%

The ICC_3,1_ was interpreted based on the Munro’s classification (very low: 0.00-0.25, low: 0.26–0.49, moderate: 0.50–0.69, high: 0.70–0.89 and very high: 0.90-1.00); A SEM% value ≤ 10% and MDC% value ≤ 30% were considered acceptable

Equations and formulas: SEM = SD√1-ICC (absolute values); SEM% = SEM / mean of measures x 100% (relative values); MDC_95_ = SEM×√2 × 1.64 (absolute values); MDC_95_% = MDC_95_ / mean of measures x 100% (relative values)

*Abbreviations:* *AD* Alzheimer’s disease, *CDR* Clinical Dementia Rating, *ICC*_3,1_ Intraclass correlation coeficiente two-way mixed model of a single-measure, *CI*_95 _95% confidence interval, *SEM *Standard error of measurement; *SEM%* Percentage of error, *MDC*_95 _Minimal detectable change at the 95% confidence interval, *MDC*_95_% Percentage of change, *n *Number of participants, $\stackrel{-}{\text{X}}$ Mean, *SD *Standard deviation, *Nm/Kg *Newton metre per kilogram, *J* Joules, *°/s *Degree per second

## Discussion

This is the first study to provide evidence of the reliability of concentric isokinetic measures of the knee and ankle muscle strength in community-dwelling older adults with AD. The reliability of the measures was also investigated in older adults without-AD. Based on the results, peak torque, average peak torque and total work are reliable measures for the assessment of knee and ankle muscle strength in community-dwelling older adults without and with AD in the mild and moderate stages.

High and very high ICC_3,1_ values were found for all isokinetic measures of the knee muscle strength at 60°/s. Therefore, the three groups investigated were similar in terms of relative reliability. Relative reliability values were similar to those reported in a previous study involving healthy older adults [13]. The knee extensor measures had lower SEM% and MDC_95_% in comparison to the flexors. SEM% values in the mild-AD and moderate-AD groups were above the cutoff point (≤ 10%). However, the mild-AD group had an acceptable MDC_95_% for average peak torque and total work. Reliable knee extensor measures are relevant, as knee extensor muscle strength is considered an important predictor of gait performance in older adults with dementia [48].

For the isokinetic measures of the knee muscle strength at 180°/s, the ICC_3,1_ values were also high or very high for all measures and similar among the three groups. However, the analysis of the means of maximum strength (peak torque) revealed that the knee flexors had better absolute reliability values. Moreover, the mild-AD group had acceptable SEM% and MDC_95_% for average peak torque of the knee flexors, which was not found in the moderate-AD group. Reliable isokinetic measures for the evaluation of knee flexors in older adults with AD is important, as isokinetic variables of knee flexor muscle strength have been found to be associated with mobility and lower limb muscle strength in healthy older adults [11]. In contrast, the analysis of the maintenance of maximum strength (total work) revealed that the knee extensors had the best absolute reliability values.

Regarding isokinetic measures of the ankle muscle strength at 30°/s, ICC_3,1_ values were high or very high for all measures in the groups without-AD and with mild-AD. However, ICC_3,1_ values for the dorsiflexors were moderate in the moderate-AD group. Thus, it seems that more advanced stages of AD exert a negative influence on the relative reliability of ankle muscle strength measures. Moreover, relative reliability of peak ankle torque in the mild-AD group was similar to that reported in a previous study involving healthy older adults [14].

The peak torque of the plantar flexors had better ICC_3,1_ values compared to the dorsiflexors in the moderate-AD group and group without-AD. This result is important, as plantar flexor muscle strength is associated with the stability of postural balance in health older adults [49]. However, a previous study involving healthy older adults found better peak ankle dorsiflexor values (30°/s) [14]. It should also be stressed that ankle muscle strength has not previously been investigated in older adults with AD.

Dorsiflexors had lower SEM% and MDC_95_% in comparison to plantar flexors in the three groups, which differs from results reported in a previous study [14]. Another important finding was that the mild-AD group had acceptable SEM% (average peak torque) and MDC_95_% (peak torque and average peak torque) for the dorsiflexors. This result indicates that a smaller change is needed to detect an improvement in these measures in interventions directed at a gain in ankle dorsiflexor muscle strength in older adults with AD. Moreover, dorsiflexor muscle strength is associated with postural balance in health older adults [50], and should therefore be evaluated in this population.

The present results can assist in the interpretation of changes in muscle strength in older adults with AD as well as the execution of future validation studies for clinical lower limb muscle strength assessment tests compared to the isokinetic measures for this population. Although the Five-times and 30-Second Sit to Stand tests are reliable for the assessment of lower limb muscle strength in older adults with AD [5], no studies have investigated the validity of these tests for this population. Concurrent validation of clinical tests with the isokinetic measures for the assessment of lower limb muscle strength has been performed for health older adults [51, 52].

## Strengths and Limitations

The strengths of the present study include the confirmation of the etiology of dementia, as only older adults with AD were included. The diagnosis of AD was performed by specialists and based on the DSM-V. The classification of the stage of AD using the CDR scale enabled the comparison between the mild and moderate stages. An isokinetic dynamometer was used with adaptation and standardization of the evaluation for older adults with AD. Lastly, a group of older adults without-AD was included for comparison purposes.

This study has some limitations to the generalization of the results. The target sample size was not reached in two groups (moderate-AD and without-AD) due to the clinical limitation of older adults, the complexity of the isokinetic evaluation, difficulties related to logistics in studies involving older adults with AD and the suspension of in-person activities at the Healthy Aging Research Laboratory and Isokinetic Dynamometry Laboratory due to the COVID-19 pandemic in March 2020. However, 91% of the sample size was reached, which is very close to the calculated number. It was not possible to blind the examiner to the different groups (mild-AD, moderate-AD and without-AD), as some instruments are administered to the caregivers to collect data on the older adults with AD. Moreover, to ensure the wellbeing and safety of the participants with AD, the caregivers were invited to participate in the evaluations.

## Conclusion

Concentric isokinetic measures are reliable for the assessment of knee and ankle muscle strength in community-dwelling older adults without and with AD in the mild and moderate stages. Relative reliability of the knee measures was similar among the different groups. In contrast, relative reliability of the dorsiflexors was lower in the group with moderate-AD. Absolute reliability of the knee extensor and ankle dorsiflexor measures was better among the groups. Despite the cognitive and physical limitations of older adults with AD and the complexity of the isokinetic evaluation, it was possible to obtain reliable measures of knee and ankle muscle strength through the adaptation and standardization of the evaluation. Future studies should define criteria of clinically relevant changes in these isokinetic measures that are applicable to older adults with AD. Validation studies are also needed to compare clinical tests to the isokinetic measures test for this population.

## Data Availability

The datasets used and/or analysed during the current study available from the corresponding author on reasonable request.

## Abbreviations

AD: Alzheimer’s disease
CDR: Clinical Dementia Rating
D1: Test
D2: Retest
DSM-V: Diagnostic and Statistical Manual of Mental Disorders
GDS: Geriatric Depression Scale
ICC: Intraclass Correlation Coefficient
MMSE: Mini Mental State Examination
MDC: Minimal Detectable Change
MBQ: Modified Baecke Questionnaire
SEM: Standard Error of Measurement
